# CollagenVI-Cre mice: A new tool to target stromal cells in secondary lymphoid organs

**DOI:** 10.1038/srep33027

**Published:** 2016-09-08

**Authors:** Alejandro Prados, George Kollias, Vasiliki Koliaraki

**Affiliations:** 1 Biomedical Sciences Research Center “Alexander Fleming”, 16672 Vari, Greece; 2 Department of Physiology, Medical School, National and Kapodistrian University of Athens, 11527 Athens, Greece

## Abstract

Stromal cells in secondary lymphoid organs (SLOs) are non-hematopoietic cells involved in the regulation of adaptive immune responses. Three major stromal populations have been identified in adult SLOs: fibroblastic reticular cells (FRCs), follicular dendritic cells (FDCs) and marginal reticular cells (MRCs). The properties of these individual populations are not clearly defined, mainly due to the lack of appropriate genetic tools, especially for MRCs. Here, we analyzed stromal cell targeting in SLOs from a transgenic mouse strain that expresses Cre recombinase under the CollagenVI promoter, using lineage tracing approaches. We show that these mice target specifically MRCs and FDCs, but not FRCs in Peyer’s patches and isolated lymphoid follicles in the intestine. In contrast, stromal cells in lymph nodes and the spleen do not express the transgene, which renders ColVI-cre mice ideal for the specific targeting of stromal cells in the gut-associated lymphoid tissue (GALT). This funding further supports the hypothesis of organ-specific stromal precursors in SLOs. Interestingly, in all tissues analyzed, there was also high specificity for perivascular cells, which have been proposed to act as FDC precursors. Taken together, ColVI-Cre mice are a useful new tool for the dissection of MRC- and FDC-specific functions and plasticity in the GALT.

The adaptive immune response is initiated in secondary lymphoid organs (SLOs), including lymph nodes (LNs), spleen and Peyer’s patches (PPs) in the intestine. These organs act as elaborate filters, located in strategic sites to maximize the chance of an encounter between lymphocytes and antigens. Despite their different macroscopic structure, they all share a complex microanatomy and the common feature of lymphocyte segregation in two different compartments, the T- and B-cell area. The T-cell area is densely populated by CD4^+^ and CD8^+^ T cells, as well as dendritic cells (DCs), while the B-cell area contains B-cells aggregated in follicles^1^. Behind this compartmentalization lies a heterogeneous population of non-hematopoietic cells that produce a variety of chemokines to attract leucocytes to each area^2^,^3^,^4^. Two major such cell populations are the most prominent: endothelial cells that are involved in the trafficking between the blood and the lymph, and stromal cells, which are responsible for the microdomain formation and maintenance of SLOs^5^,^6^.

During embryonic development, stromal cells in SLOs originate from mesenchymal precursors^7^,^8^ which interact with hematopoietic lineage cells to induce a differentiation program^9^. First, mesenchymal precursors are differentiated into lymphoid tissue organizer cells (LTo cells) through interactions with lymphoid tissue inducer cells (LTi cells). Later, B and T cells induce the differentiation of LTo cells in at least three subpopulations: fibroblastic reticular cells (FRCs) in the T-cell area, follicular dendritic cells (FDCs) in the B-cell area and marginal reticular cells (MRCs) in the SLO periphery^2^,^10^. FRCs play a crucial role in T cell maintenance through the production of survival factors, such as IL-7^11^, in the guidance of T cell and DC migration through CCL19 and CCL21 secretion^3^ and in the formation of a microvascular conduit system that distributes small antigens within SLOs^12^. Similarly, FDCs are important for the B-cell area maintenance through the production of B cell survival factors, such as IL-15 or BAFF^13^,^14^, the guidance of B cell migration through CXCL12 and CXCL13^15^,^16^ and the facilitation of high-affinity antibody production in germinal centers^17^. Finally, MRCs are the most recent stromal cell population described^18^ and they are still poorly characterized. Jarjour *et al*., however, recently showed that MRCs can function as FDC precursors in LNs^19^. Besides FRCs, FDCs and MRCs, which are the major stromal populations in adult SLOs, additional stromal cell types are also present in virtually all these tissues. These include cells surrounding blood and lymphatic vessels, generally called pericytes, which have important functions in vascular morphogenesis, hemostasis, and lymph propulsion^20^,^21^. The precise origin of these cells, as well as the relationship between them and other stromal cell types in SLOs is not clearly defined.

The elucidation of the origin, properties and functions of individual cell populations is facilitated by the use of appropriate genetic tools for their specific manipulation. The development of the Cre-LoxP system has provided such a powerful tool in combination with genetic targeting and cell lineage tracing approaches. This technology is based on the expression of the bacteriophage P1 Cre-recombinase under the control of cell type-specific promoters^22^. In the case of SLOs, the most common genetic tools used for the study of SLO stromal cells include the CD21-Cre mice that target FDCs in all SLOs, the PDPN-Cre mice that target FRCs in LNs and CCL19-Cre mice that target FRCs in all SLOs^23^,^24^,^25^,^26^. These strains, however, show also some specificity for other non-stromal populations, such as B cells^23^, endothelial cells^24^ or epithelial cells^27^, while there is no genetic tool to target MRCs to date.

In this study, we used a transgenic mouse strain that expresses Cre-recombinasese under the CollagenVI promoter (ColVI-Cre mice) in combination with cell lineage approaches. We show that ColVI-Cre mice specifically target MRCs and FDCs, but not FRCs in PPs. We also demonstrate that FDCs and MRCs in other SLOs are not targeted, with the exception of a small fraction in peripheral lymph nodes (pLNs). Finally, we show that ColVI-Cre mice target pericytes around blood, but not lymphatic, vessels in all SLOs tested. ColVI-cre mice could therefore, facilitate the analysis of MRC- and FDC-specific functions and plasticity in the gut-associated lymphoid tissue (GALT).

## Results

### ColVI-Cre mice target FDCs and MRCs in PPs

We have previously demonstrated that ColVI-Cre transgenic mice show specificity for mesenchymal cells in the intestine, synovium and skin^28^,^29^. Since FDCs and FRCs can originate from myofibroblastic precursors^7^,^25^ we analyzed the specificity of this mouse strain for stromal populations in different SLOs. To accomplish this, we crossed ColVI-Cre mice to Rosa26^mT/mG^ reporter mice^30^. These mice normally express the membrane-targeted Tomato protein (mT), which is excised after Cre-mediated recombination, allowing thus for the expression of the membrane-targeted green fluorescent protein (mG).

**Figure 1:**
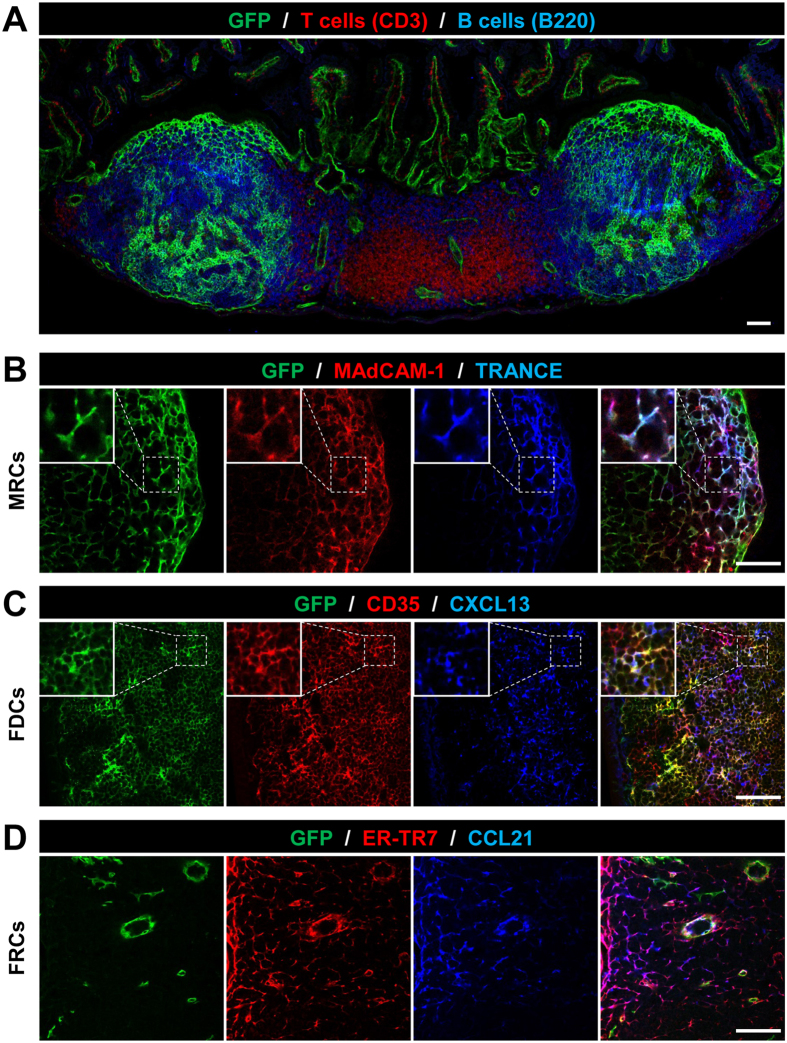
ColVI-Cre^+^ cells are present in PPs and co-localize with MRC- and FDC- specific markers. (**A**) Representative image of a PP from ColVI–Cre, R26^mT/mG^ mice stained for T cells (CD3 in red), B cells (B220 in blue) and Cre-mediated GFP expression. (**B–D**) Co-localization of Cre-mediated GFP expression with stromal cell markers. MRCs are marked by MAdCAM-1 and TRANCE (**B**), FDCs by CD35 and CXCL13 (**C**) and FRCs by ER-TR7 and CCL21 (**D**). Inserts display higher magnification of selected areas. Scale bars, 75 μm. Several PPs from 5 different transgenic mice were analyzed.

Confocal laser-scanning microscopy of PPs from ColVI-Cre × Rosa26^mT/mG^ revealed expression of the ColVI-Cre transgene mainly in stromal cells within the B-cell area and only in a few cells of the T-cell area (Fig. 1A). To better characterize the pattern of the transgene expression, PP sections were further stained with a combination of markers, so as to identify FDC, MRC, and FRC-specific networks. GFP positive MRCs were identified as TRANCE^+^ and MAdCAM-1^+^ cells located under the epithelial dome (Fig. 1B). FDCs, detected near the muscularis layer as CD35^+^ and CXCL13^+^ cells, also exhibited high GFP expression (Fig. 1C). In contrast, FRCs, identified as cells expressing ERTR7 and CCL21 in the T cell area, were GFP negative (Fig. 1D).

**Figure 2:**
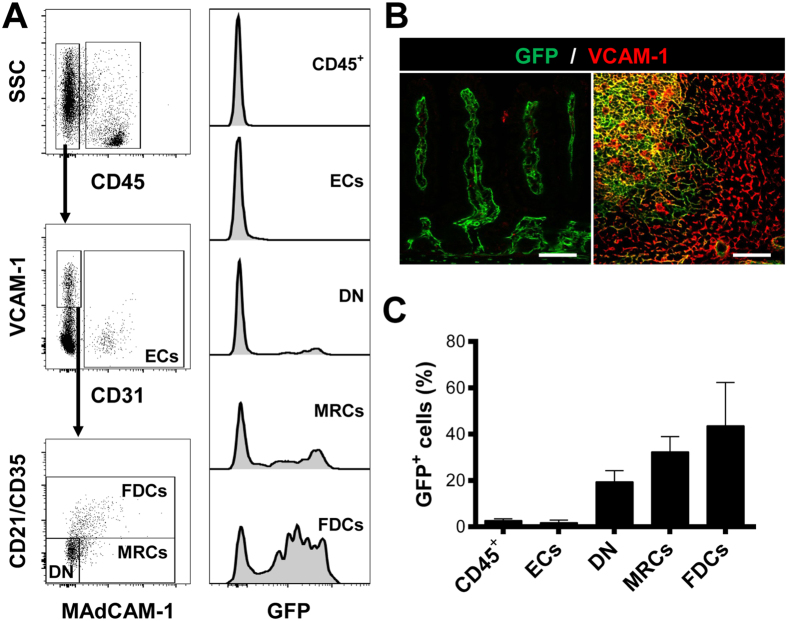
ColVI-Cre targets approximately 50% of FDCs and 35% of MRCs in PPs. (**A**) Representative FACS analysis of digested PPs enriched in CD45 negative cells. Hematopoietic lineage cells were identified as CD45^+^ cells and ECs as CD45^−^ CD31^+^ cells. SLO stromal cells (CD45^−^ CD31^−^ VCAM-1^+^ cells) were divided in three subpopulations: FDCs (CD35^+^), MRCs (MAdCAM-1^+^ CD35^−^) and double negative cells (DN: MAdCAM-1^−^ CD35^−^). Right histograms show GFP expression in every population. (**B**) Representative images of mouse small intestine and PPs stained with VCAM-1. Scale bars, 75 μm. (**C**) Quantification of the percentage of GFP^+^ cells in the populations established in (**A**). Data are presented as mean ± SE (n = 4 mice).

To quantify the transgene expression in PPs, we next performed FACS analysis. Flow cytometry showed that GFP expression was specific for the stromal compartment, since hematopoietic (CD45^+^) and endothelial cells (CD45^−^CD31^+^) were not targeted by ColVI-Cre mice (Fig. 2A), in agreement to what has been previously published^28^,^29^. To discriminate between PP stromal cells and intestinal mesenchymal cells, which are also ColVI-Cre^+^, we used VCAM-1, an adhesion molecule only expressed by PP stromal cells under homeostasis (Fig. 2B). PP stromal cells, gated as CD45^−^CD31^−^VCAM-1^+^ cells, were divided then in three subpopulations: FDCs as CD21/CD35^+^ cells, MRCs as CD21/CD35^−^ MAdCAM-1^+^ cells and double negative (DN) cells as CD21/CD35^−^ MAdCAM-1^−^ cells. FACS analysis revealed that approximately 50% of FDCs and 35% of MRCs were GFP^+^ (Fig. 2A,C). This was in agreement with the confocal imaging data, as GFP negative areas were noticeable inside FDC and MRC networks (Fig. 1B,C). Moreover, we detected approximately 20% of DN cells, which were also GFP^+^. DN cells are usually composed by different stromal population, including FRCs, pericytes and other poorly characterized subpopulations from the B cell area, such as CXCL12-expressing reticular cells (CRCs) or follicular stromal cells (FSCs), both of which are negative for CD21/CD35 and MAdCAM-1 15,31. These results suggest that ColVI-Cre mice can be a unique tool for the specific targeting of FDCs and MRCs, but not FRCs, in PPs without any specificity for non-stromal cell types. Moreover, their potential specificity for other under-represented stromal populations in the B-cell area could prove important for their further characterization.

### ColVI-Cre mice target stromal cells in isolated lymphoid follicles in the intestine

**Figure 3:**
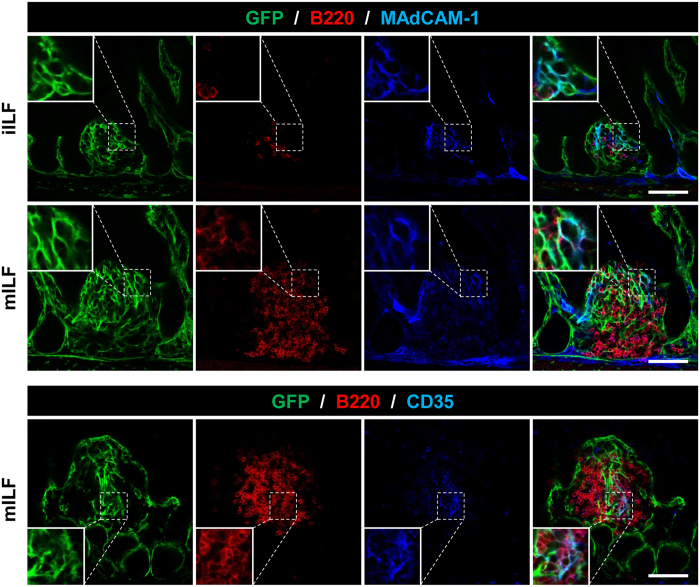
ColVI-Cre targets MRCs and FDCs in ILFs. Representative images of immature (iILFs) and mature ILFs (mILFs), showing Cre-mediated GFP expression. B cells are marked as B220^+^ cells, MRCs as MAdCAM-1^+^ networks, and FDCs as CD35^+^ networks. Inserts display higher magnification of selected areas. Scale bars, 75 μm. Several ILFs from 5 different transgenic mice were analyzed.

PPs are part of the GALT along with isolated lymphoid follicles (ILFs). ILFs were described relatively recently as single B-cell aggregates with an architecture and cellular composition similar to PPs^32^,^33^. They develop postnatally and their size and composition depends on external stimuli and commensal bacteria, producing a spectrum of appearances ranging from small B cell clusters (immature ILFs) to well-organized lymphoid structures containing germinal centers (mature ILFs)^34^. Apart from B and T-cells they also include stromal cells, which are still poorly characterized. Using ColVI-Cre-Rosa26^mT/mG^ mice, we further identified GFP expression inside both immature and mature ILFs, which co-localized with MAdCAM-1 and CD35 expression, marking MRCs and FDCs, respectively (Fig. 3). We also observed a continuous GFP^+^ network that connects the intestinal and ILF stroma, suggesting a close relationship between the two cell populations. Together, these results indicate that ColVI-Cre mice may be used to target particular stromal subsets in the GALT and could help to elucidate both their specific functions and their potential ontogenetic relationships.

### ColVI-Cre mice do not show specificity for stromal cells in mesenteric LNs or the spleen

**Figure 4:**
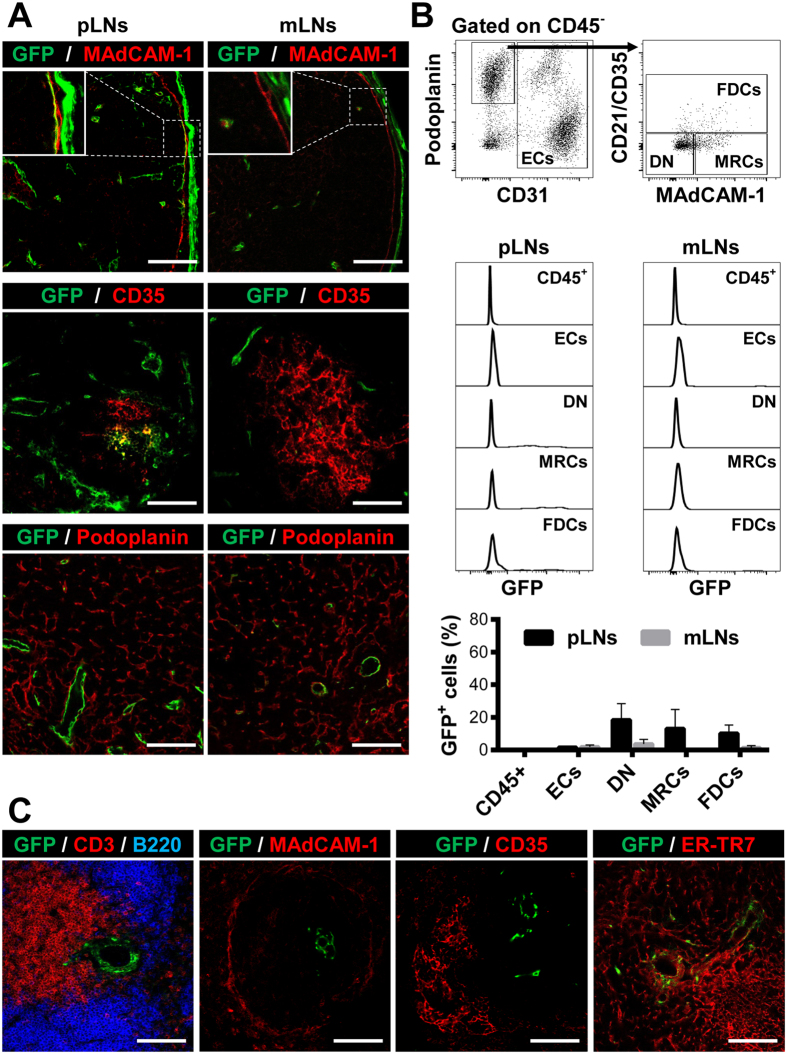
ColVI-Cre^+^ cells are absent in LNs and the spleen. (**A**) Representative images of pLNs and mLNs showing Cre-mediated GFP expression. MRCs are marked as MAdCAM-1^+^ networks, FDCs as CD35^+^ cells and FRCs as podoplanin^+^ networks. (**B**) Representative FACS analysis of digested LNs from ColVI-Cre-R26^mT/mG^ mice showing gating strategy and GFP expression in individual cell populations. Graph shows the quantification of the GFP^+^ cell percentage in the different populations. Data are presented as mean ± SE (n = 4 mice). (**C**) Representative images of spleen from ColVI-Cre-R26^mT/mG^, stained with antibodies against CD3 and B220, for T and B cells, respectively. MRCs are marked by MAdCAM-1, FDCs by CD35 and FRCs by ER-TR7. Inserts display higher magnification of selected areas. Scale bar, 75 μm. 5 different mice were analyzed.

Applying similar immunofluorescent techniques, we also analyzed pLNs and more specifically, inguinal and axillar LNs, as well as mesenteric LNs (mLNs). In contrast to PPs, we could mainly detect transgene activity in the collagen capsule, but not in stromal cells in either the T-cell or B-cell area, as shown both by confocal microscopy (Fig. 4A) and FACS analysis (Fig. 4B). GFP^+^ cells were especially rare in mLNs (Fig. 4B), while in pLNs only a few FDCs and MRCs were GFP positive (Fig. 4A,B). In both cases, we could not detect GFP expression in the FRC network, marked as podoplanin positive cells.

Accordingly, similar analysis of the spleen showed that GFP^+^ cells were equally rare and only a few cells could be detected in the central area of the white pulp (Fig. 4C). Again, co-staining with stromal specific markers and analysis by confocal microscopy showed lack of GFP expression by FDCs, MRCs or FRCs. These results suggest that, in contrast to PPs, other SLOs, including pLNs, mLNs and the spleen, are not targeted by the ColVI-Cre mouse strain (Table 1), which may allow for the analysis of local SLO stromal cell functions in the intestine, independently of relevant functions of SLOs in the periphery and especially closely related mLNs. It also further supports the hypothesis of distinct organ-specific stromal cell precursors in the different SLOs^8^.

### Pericytes are targeted by ColVI-Cre in all SLOs

**Figure 5:**
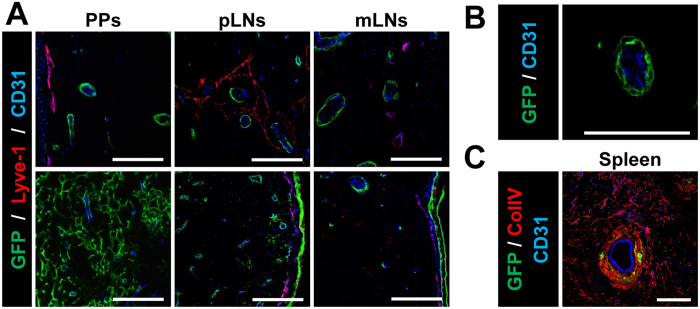
ColVI-Cre mice target perivascular cells in SLOs. (**A**) Representative images of T- (top) and B- (bottom) cell areas in PPs, pLNs and mLNs, showing Cre-mediated GFP expression in relation to blood and lymphatic vessels. BECs are marked as CD31^+^ cells (blue) and LECs as CD31^+^ Lyve-1^+^ cells (purple). (**B**) Higher magnification image of a blood vessel (CD31^+^) and GFP^+^ surrounding it. (**C**) Spleen image showing GFP^+^ cells only in the central part of the white pulp, surrounding endothelial cells (CD31^+^ cells in blue) and co-localizing with collagen IV (red). Scale bar, 75 μm. 5 different mice were analyzed.

During the previous experiments, we noticed that GFP^+^ cells could also be found around vessels in all SLOs analyzed. To identify whether ColVI-Cre^+^ cells were found around both blood and lymphatic vessels, we stained tissues with CD31, a common marker for blood (BECs) and lymphatic endothelial cells (LECs), and LYVE-1, which is specific only for LECs. In PPs and LNs, GFP^+^ cells were found surrounding CD31-positive endothelial cells in both T and B cell areas, but not LYVE-1^+^ LECs (Fig. 5A,B). In the spleen, high-resolution analysis revealed that GFP^+^ cells were mainly located surrounding CD31^+^ cells in the central arteriole (Fig. 5C). These data indicate different properties and origin for blood and lymphatic pericytes in SLOs, and ColVI-Cre mice could be a useful new tool to discern the specific role of pericytes in cell trafficking regulation between blood and SLOs.

## Discussion

In this study, we analyzed the cell-specificity of the ColVI-Cre transgenic mouse strain in SLOs, including the spleen, pLNs, mLNs and PPs in the intestine, in addition to the known specificities for mesenchymal cells in the intestine, synovium and skin^28^,^29^. We show that ColVI-Cre mice do not show any specificity for hematopoietic, epithelial or endothelial cell lineages and that cre recombinase specificity is confined to the GALT stroma. We further show that ColVI-Cre marks FDCs and MRCs in PPs, as well as stromal cells in ILFs, while they show no specificity for FRCs and stromal cells in other SLOs. Furthermore, it is the first strain that to our knowledge specifically targets MRCs *in vivo*, offering, thus new possibilities for the functional characterization of this cell population during development and in immunity. It should be noted that the additional specificities of the ColVI-cre mouse, especially for other mesenchymal cells in the intestine, might complicate targeting strategies when used with floxed mice and caution is needed in the interpretation of results. However, we believe that it is still a valuable tool for the study of specific stromal cell function in the GALT, independently of relevant stromal cell function in other SLOs and particularly closely related mLNs.

PPs, LNs and the white pulp of the spleen bear developmental, cellular and molecular similarities. According to the current SLO developmental model, FDCs, MRCs and FRCs originate from a common mesenchymal precursor^7^,^8^,^9^; however, it is likely to be a different one depending on the organ. In fact, an organ-specific stromal precursor has been identified in the spleen, and it does not play a role in the development of the LN and PP stroma^8^. Our results further support this hypothesis, since we have detected ColVI-Cre expression only in stromal networks in PPs and ILFs, but not in mLNs or the spleen. The presence of a few GFP^+^ cells in pLNs may also point to the existence of several stromal precursors in LNs and it is now well-established that pLNs and mLNs have different developmental requirements^9^ and differences in their respective stromal precursor have been reported^35^.

Mesenchymal precursor differentiation into mature stromal cells happens in several steps. First, precursors are differentiated into LTo cells, defined by high ICAM-1 and VCAM-1 expression and by the upregulation of essential molecules involved in organogenesis, including CCL19, CXCL13 or TRANCE^36^,^37^. Later, when lymphocytes arrive into the SLO primordium, LTo cells are differentiated into FRCs, FDCs or MRCs, depending on the lymphoid region^38^,^39^. The connection between these populations and LTo cells is still controversial, with some reports suggesting a close developmental relationship between FRCs and MRCs^40^, while others have shown a different developmental pathway using the CCL19-Cre mice^25^. Our work is in agreement with Chai *et al*., and further supports the hypothesis that there are different precursors for FRCs and MRCs at least in PPs, since ColVI-Cre mice target MRCs, but not FRCs. The relation between MRCs and FDCs seems to be more clear, since a recent paper has corroborated that MRCs can act as a source of FDCs in LNs^19^. Similarly, in the ColVI-Cre mice, both cell types showed reporter gene expression, which indicates an ontogenic relationship between the two cells types in PPs, although we cannot exclude that adult PP MRCs and FDCs use Collagen VI gene and, as consequence, both of them express GFP, under the control of the ColVI promoter. It is also obvious from our studies that ColVI-Cre mice do not target the 100% of either MRCs or FDCs. This indicates that ColVI-Cre^+^ cells are not early progenitors and that they probably appear in later developmental stages or that more than one cell types can act as PP stromal cell progenitor cells, which needs to be further analyzed through experiments in the PP anlangen.

Besides FDCs and MRCs in the B cell area, other subpopulations have also been described, such as CRCs or FSCs^15^,^31^. However, these cell types are poorly characterized mainly due to the lack of specific cell surface markers. Interestingly, the ColVI-Cre mouse line displayed a transgene expression pattern that could indicate its expression in these stromal cell subpopulations, since we have noted the presence of GFP^+^CD35^−^MAdCAM-1^−^ cells near to the muscular layer and between FDC and MRC networks, where CRCs and FSCs are respectively located and it could therefore facilitate their characterization. GFP^+^CD35^−^MAdCAM-1^−^ cells were also identified by FACS. A relationship between these rare stromal cell populations and FRCs has previously been suggested due to phenotypic similarities and their common targeting by CCL19-Cre mice in LNs and PPs^31^,^41^. However, our data suggest that at least in PPs, there is a common origin only for stromal cells in the B cell area and not FRCs. Future experiments using multi-fluorescent reporter mice are required to further clarify the cell ontogeny in the different SLOs.

It has also been recently demonstrated that spleen FDCs originate from a perivascular precursor^7^. This location is occupied by different stromal cell types, such as vascular smooth muscle cells (vSMCs), fibroblasts or pericytes. However, there is no single molecular marker known to distinguish them^20^. Here, we demonstrated that ColVI-Cre mice also target perivascular cells in all SLOs, including the spleen. Nevertheless, this population should be different from the FDC precursor, described by Krautler *et al*., since we could not identify GFP expression in spleen FDCs. Similar results have been also shown by Onder *et al*., who have identified a perivascular cell type targeted by PDPN-Cre mice, which does not act as a precursor of FDCs in the spleen^24^. Taking into consideration that these cells are not involved in the formation of the stroma, they could have a role in the maintenance of endothelial venule integrity^42^.

The finding that ColVI-Cre mice also label stromal cells in ILFs offers a first indication that tertiary lymphoid structures (TLOs) could also be targeted in this mouse strain. In contrast to the spleen, LNs and PPs, ILFs are inducible structures developed after birth due to the presence of external stimuli^43^. They are widely associated with infection and chronic inflammation, however, their role in these pathologies is still controversial, as they can either contribute to the local protective immune response or support disease progression^44^. It would, therefore be interesting to further examine tertiary lymphoid organogenesis in this mouse strain, both in the intestine and the peripheral SLOs and discern the ontogenic and functional relationships between pericytes, mesenchymal stromal and lymphoid stromal cells under such conditions.

In summary, the ColVI–Cre mouse model is a promising new tool to target stromal cells in the B cell area and the first one that targets MRCs in the GALT. We believe that this transgenic line could be a valuable tool for the manipulation of gene expression in the follicular stroma without affecting stromal cell lineages in other SLOs and for the clarification of the relationship between them and the rest of SLO stromal cells. Ultimately, this could assist in the elucidation of stromal-immune interaction both in homeostasis and in disease.

## Materials and Methods

### Mice

The generation of ColVI-Cre mice has been previously described^28^. ColVI-Cre mice were crossed with R26^mT/mG^ reporter mice^30^ to analyze Cre-recombinase expression. All mice were bred and maintained on a C57BL/6J genetic background in the animal facilities of the Biomedical Sciences Research Center “Alexander Fleming” under specific pathogen-free conditions. Experiments were approved by the Institutional Committee of Protocol Evaluation in conjunction with the Veterinary Service Management of the Hellenic Republic Prefecture of Attika according to all current European and national legislation and were performed in accordance with relevant guidelines and regulations.

### Immunohistochemistry

LNs, spleens and small intestines containing PPs were harvested, washed with PBS, fixed in 4% PFA for 1 hour and snap-frozen in an OCT-filled mold on a liquid nitrogen-cooled metal surface. Cryosections 10 μm were rehydrated in wash buffer (0.1% saponin in PBS) for 5 min and blocked in PBS containing 0.5% albumin for 30 min. Sections were incubated with antibodies against: collagen IV and ER-TR7 from Abcam; TRANCE, eFluor 660-conjugated anti-podoplanin and anti-Lyve1 from eBioscience; CXCL13 and CCL21 from R&D Systems; CD31 and CD35 from BD Pharmigen; MAdCAM-1, Alexa Fluor 594-conjugated anti-CD3 and Alexa Fluor 647-conjugated anti-B220 from Biolegend; and Alexa Fluor 488-conjugated anti-GFP from Invitrogen. Unconjugated antibodies were detected with the following secondary antibodies: Alexa Fluor 594-conjugated anti-rat-IgG, Alexa Fluor 568-conjugated anti-rabbit-IgG all purchased from Invitrogen. TRANCE, CXCL13 and CCL21 expression were detected using the tiramide amplification signal kit (Applied biosystem). Finally, images were acquired with a TCS SP8X White Light Laser confocal system (Leica).

### Stromal cell isolation

PPs and LNs were harvested and washed with cold PBS. Epithelial cells from PPs were eliminated after incubation in Hank’s balanced salt solution (HBSS) supplemented with 5 mM EDTA, 1mM DTT and 20 μM HEPES for 30 minutes at 37 °C. Then, both PPs and LNs were dissected into small pieces and digested using an enzyme mix comprised of DMEM medium containing 0.8 mg/ml Dispase, 0.1 mg/ml Collagenese P (Roche) and 0.1 mg/ml DNase I (Sigma) for 50 min at 37 °C, as it was described previously^45^. To further enrich the stromal cell fraction, lymphocytes were depleted by incubating the cell suspension with MACS anti-CD45 microbeads and passing over a MACS LS column (Miltenyi Biotec). The cells in the flow-through were collected and analyzed by flow cytometry. Viability, assessed by trypan blue exceeded 90%.

### Flow cytometry analysis

Stromal cells were suspended in FACS buffer (PBS supplemented with 0.5% bovine serum albumin (BSA) and 5 mM EDTA in PBS) at 10^7^ cells/ml and blocked using 4% of normal mouse serum. 100 μl of cell suspension was stained for 30 min at 4 °C using the following antibodies: PerCP-conjugated anti-CD31 (clone 390), PE-Cy7-conjugated anti-VCAM-1, PE-Cy7-conjugated anti-podoplanin, Alexa Fluor 700 conjugated anti-CD45, APC-Cy7-conjugated anti-CD21/CD35 and anti-MAdCAM-1 (Biolegend). Alexa Fluor 647-conjugated anti-rat-IgG (Invitrogen) was used as a secondary antibody to detect MAdCAM-1 expression. Finally, cells were acquired on a FACSCANTO II (BD Bioscience) and data were analyzed using Flowjo software (Tree star Inc).

## Additional Information

**How to cite this article**: Prados, A. *et al*. CollagenVI-Cre mice: A new tool to target stromal cells in secondary lymphoid organs. *Sci. Rep.* **6**, 33027; doi: 10.1038/srep33027 (2016).

## Figures and Tables

**Table 1:** Populations targeted by ColVI-Cre mice in the different SLOs.

	PPs	pLNs	mLNs	Spleen
FRCs	−	−	−	−
FDCs	+	+/−	−	−
MRCs	+	+/−	−	−
Pericytes	+	+	+	+/−

PPs, Peyer’s Patches; pLNs, peripheral lymph nodes; mLNs, mesenteric lymph nodes; FRCs, fibroblastic reticular cells; FDCs, follicular dendritic cells; MRCs, marginal reticular cells.

## References

[b1] Junt T., Scandella E. & Ludewig B. Form follows function: lymphoid tissue microarchitecture in antimicrobial immune defence. Nat. Rev. Immunol. 8, 764–775 (2008). 18825130 10.1038/nri2414

[b2] Buettner M., Pabst R. & Bode U. Stromal cell heterogeneity in lymphoid organs. Trends Immunol. 31, 80–86 (2010). 19969504 10.1016/j.it.2009.11.003

[b3] Bajénoff M. . Stromal cell networks regulate lymphocyte entry, migration, and territoriality in lymph nodes. Immunity 25, 989–1001 (2006). 17112751 10.1016/j.immuni.2006.10.011 PMC2692293

[b4] Weninger W. . Naive T Cell Recruitment to Nonlymphoid Tissues: A Role for Endothelium-Expressed CC Chemokine Ligand 21 in Autoimmune Disease and Lymphoid Neogenesis. J. Immunol. 170, 4638–4648 (2003). 12707342 10.4049/jimmunol.170.9.4638

[b5] Koning J. J. & Mebius R. E. Interdependence of stromal and immune cells for lymph node function. Trends Immunol. 33, 264–270 (2012). 22153930 10.1016/j.it.2011.10.006

[b6] Buettner M. & Bode U. Stromal cells directly mediate the re-establishment of the lymph node compartments after transplantation by CXCR5 or CCL19/21 signalling. Immunology 133, 257–269 (2011). 21426341 10.1111/j.1365-2567.2011.03436.x PMC3088987

[b7] Krautler N. J. . Follicular dendritic cells emerge from ubiquitous perivascular precursors. Cell 150, 194–206 (2012). 22770220 10.1016/j.cell.2012.05.032 PMC3704230

[b8] Castagnaro L. . Nkx2-5(+)islet1(+) mesenchymal precursors generate distinct spleen stromal cell subsets and participate in restoring stromal network integrity. J. Immunol. 38, 782–791 (2013). 10.1016/j.immuni.2012.12.005 PMC3652017 23601687

[b9] van de Pavert S. a. & Mebius R. E. New insights into the development of lymphoid tissues. Nat. Rev. Immunol. 10, 664–674 (2010). 20706277 10.1038/nri2832

[b10] Roozendaal R. & Mebius R. E. Stromal Cell–Immune Cell Interactions. Annu. Rev. Immunol. 29, 23–43 (2011). 21073333 10.1146/annurev-immunol-031210-101357

[b11] Link A. . Fibroblastic reticular cells in lymph nodes regulate the homeostasis of naive T cells. Nat. Immunol. 8, 1255–1265 (2007). 17893676 10.1038/ni1513

[b12] Gretz J. E., Norbury C. C., Anderson A. O., Proudfoot A. E. I. & Shaw S. Lymph-Borne Chemokines and Other Low Molecular Weight Molecules Reach High Endothelial Venules via Specialized Conduits While a Functional Barrier Limits Access to the Lymphocyte Microenvironments in Lymph Node Cortex. J. Exp. Med. 192, 1425–1440 (2000). 11085745 10.1084/jem.192.10.1425 PMC2193184

[b13] Rahman Z. S. M. & Manser T. B Cells Expressing Bcl-2 and a Signaling-Impaired BAFF-Specific Receptor Fail to Mature and Are Deficient in the Formation of Lymphoid Follicles and Germinal Centers. J. Immunol. 173, 6179–6188 (2004). 15528355 10.4049/jimmunol.173.10.6179

[b14] Cui G. . Characterization of the IL-15 niche in primary and secondary lymphoid organs *in vivo*. Proc. Natl. Acad. Sci. 111, 1915–1920 (2014). 24449915 10.1073/pnas.1318281111 PMC3918838

[b15] Allen C. D. C. & Cyster J. G. Follicular dendritic cell networks of primary follicles and germinal centers: phenotype and function. Semin. Immunol. 20, 14–25 (2008). 18261920 10.1016/j.smim.2007.12.001 PMC2366796

[b16] Allen C. D. C. . Germinal center dark and light zone organization is mediated by CXCR4 and CXCR5. Nat. Immunol. 5, 943–952 (2004). 15300245 10.1038/ni1100

[b17] El Shikh M. E. M., El Sayed R. M., Sukumar S., Szakal A. K. & Tew J. G. Activation of B cells by antigens on follicular dendritic cells. Trends Immunol. 31, 205–211 (2010). 20418164 10.1016/j.it.2010.03.002 PMC2886728

[b18] Katakai T. . Organizer-like reticular stromal cell layer common to adult secondary lymphoid organs. J. Immunol. 181, 6189–6200 (2008). 18941209 10.4049/jimmunol.181.9.6189

[b19] Jarjour M. . Fate mapping reveals origin and dynamics of lymph node follicular dendritic cells. J. Exp. Med. 211, 1109–1122 (2014). 24863064 10.1084/jem.20132409 PMC4042641

[b20] Armulik A., Genové G. & Betsholtz C. Pericytes: Developmental, Physiological, and Pathological Perspectives, Problems, and Promises. Dev. Cell 21, 193–215 (2011). 21839917 10.1016/j.devcel.2011.07.001

[b21] Tammela T. & Alitalo K. Lymphangiogenesis: Molecular Mechanisms and Future Promise. Cell 140, 460–476 (2010). 20178740 10.1016/j.cell.2010.01.045

[b22] Wang X. Cre transgenic mouse lines. Methods Mol. Biol. 561, 265–273 (2009). 19504077 10.1007/978-1-60327-019-9_17

[b23] Kraus M., Alimzhanov M. B., Rajewsky N. & Rajewsky K. Survival of Resting Mature B Lymphocytes Depends on BCR Signaling via the Igα/β Heterodimer. Cell 117, 787–800 (2004). 15186779 10.1016/j.cell.2004.05.014

[b24] Onder L. . A novel bacterial artificial chromosome-transgenic podoplanin-cre mouse targets lymphoid organ stromal cells *in vivo*. Front. Immunol. 2, 50 (2011). 22566840 10.3389/fimmu.2011.00050 PMC3342134

[b25] Chai Q. . Maturation of Lymph Node Fibroblastic Reticular Cells from Myofibroblastic Precursors Is Critical for Antiviral Immunity. Immunity 38, 1013–1024 (2013). 23623380 10.1016/j.immuni.2013.03.012 PMC7111182

[b26] Victoratos P. . FDC-specific functions of p55TNFR and IKK2 in the development of FDC networks and of antibody responses. Immunity 24, 65–77 (2006). 16413924 10.1016/j.immuni.2005.11.013

[b27] Onder L. . Alternative NF-κB signaling regulates mTEC differentiation from podoplanin-expressing presursors in the cortico-medullary junction. Eur. J. Immunol. 45, 2218–2231 (2015). 25973789 10.1002/eji.201545677

[b28] Armaka M. . Mesenchymal cell targeting by TNF as a common pathogenic principle in chronic inflammatory joint and intestinal diseases. J. Exp. Med. 205, 331–337 (2008). 18250193 10.1084/jem.20070906 PMC2271010

[b29] Koliaraki V., Pasparakis M. & Kollias G. IKKβ in intestinal mesenchymal cells promotes initiation of colitis-associated cancer. J. Exp. Med. 212, 2235–2251 (2015). 26621453 10.1084/jem.20150542 PMC4683996

[b30] Muzumdar M., Tasic B., Miyamichi K., Li L. & Luo L. A global double fluorescent Cre reporter mouse. Genesis 605, 593–605 (2007). 17868096 10.1002/dvg.20335

[b31] Rodda L. B., Bannard O., Ludewig B., Nagasawa T. & Cyster J. G. Phenotypic and Morphological Properties of Germinal Center Dark Zone Cxcl12-Expressing Reticular Cells. J. Immunol. 195, 4781–4791 (2015). 26453751 10.4049/jimmunol.1501191 PMC4637241

[b32] Moghaddami M., Cummins A. & Mayrhofer G. Lymphocyte-filled villi: comparison with other lymphoid aggregations in the mucosa of the human small intestine. Gastroenterology 115, 1414–1425 (1998). 9834269 10.1016/s0016-5085(98)70020-4

[b33] Hamada H. . Identification of Multiple Isolated Lymphoid Follicles on the Antimesenteric Wall of the Mouse Small Intestine. J. Immunol. 168, 57–64 (2002). 11751946 10.4049/jimmunol.168.1.57

[b34] Lorenz R. G., Chaplin D. D., McDonald K. G., McDonough J. S. & Newberry R. D. Isolated Lymphoid Follicle Formation Is Inducible and Dependent Upon Lymphotoxin-Sufficient B Lymphocytes, Lymphotoxin Receptor, and TNF Receptor I Function. J. Immunol. 170, 5475–5482 (2003). 12759424 10.4049/jimmunol.170.11.5475

[b35] Cupedo T. . Presumptive lymph node organizers are differentially represented in developing mesenteric and peripheral nodes. J. Immunol. 173, 2968–2975 (2004). 15322155 10.4049/jimmunol.173.5.2968

[b36] Withers D. R. . The role of lymphoid tissue inducer cells in splenic white pulp development. Eur. J. Immunol. 37, 3240–3245 (2007). 17948268 10.1002/eji.200737541

[b37] Bénézech C. . Ontogeny of stromal organizer cells during lymph node development. J. Immunol. 184, 4521–4530 (2010). 20237296 10.4049/jimmunol.0903113 PMC2862734

[b38] Cupedo T. . Initiation of cellular organization in lymph nodes is regulated by non-B cell-derived signals and is not dependent on CXC chemokine ligand 13. J. Immunol. 173, 4889–4896 (2004). 15470030 10.4049/jimmunol.173.8.4889

[b39] Bajénoff M. & Germain R. N. B-cell follicle development remodels the conduit system and allows soluble antigen delivery to follicular dendritic cells. Blood 114, 4989–4997 (2009). 19713459 10.1182/blood-2009-06-229567 PMC2788973

[b40] Katakai T. Marginal reticular cells: a stromal subset directly descended from the lymphoid tissue organizer. Front. Immunol. 3, 1–6 (2012). 22807928 10.3389/fimmu.2012.00200 PMC3395019

[b41] Cremasco V. . B cell homeostasis and follicle confines are governed by fibroblastic reticular cells. Nat. Immunol. 15, (2014). 10.1038/ni.2965 PMC4205585 25151489

[b42] Herzog B. H. . Podoplanin maintains high endothelial venule integrity by interacting with platelet CLEC-2. Nature 502, 105–109 (2013). 23995678 10.1038/nature12501 PMC3791160

[b43] Eberl G. & Lochner M. The development of intestinal lymphoid tissues at the interface of self and microbiota. Mucosal Immunol. 2, 478–485 (2009). 19741595 10.1038/mi.2009.114

[b44] Lochner M. Tertiary lymphoid tissues in the colon: Friend and foe. http://dx.doi.org/10.4161/gmic.2.3.16732 (2011). 10.4161/gmic.2.3.16732 21869608

[b45] Fletcher A. L. . Reproducible isolation of lymph node stromal cells reveals site-dependent differences in fibroblastic reticular cells. Front. Immunol. 2, 35 (2011). 22566825 10.3389/fimmu.2011.00035 PMC3342056

